# On Syphilitic Epilepsy

**Published:** 1871-09

**Authors:** Reuben A. Vance

**Affiliations:** Attending Physician for Diseases of the Nervous System at the Out-Door Department of Bellevue Hospital


### Selections.

*On Syphilitic Epilepsy.* By Reuben A. Vance, M.D., Attending Physician for Diseases of the Nervous System at the Out-Door Department of Bellevue Hospital.

At the present time physicians group together under the general name of epilepsy, conditions as widely unlike as the imperceptible nervous changes induced by the unfelt irritation of a slight peripheral lesion and the grave disorganization produced by a tumor of the brain. With the advance of pathological knowledge we shall doubtless see this nomenclature disappear, and have in its place one more consonant with the present state of our knowledge of the physiology of the nervous system. The symptoms embraced under this name are as discordant as the pathological conditions which produce them. The merest sense of vertigo is as truly epileptic as the most completely developed paroxysm ; and between these two extremes the disease presents almost numberless varieties. Dr. Russell Reynolds insists that the essential elements of the disease are loss or trouble of consciousness and excess of muscular contraction — that the former may be the only one apparent, the latter being limited to over-exertion of the muscular walls of the cerebral vessels.

It is a matter of common observation that symptoms of this nature are developed during the course of many different diseases. The convulsions of infancy, the eclampsia of parturient women, and the spasms and unconsciousness attending the progress of cerebral diseases, are cases in point. The phenomena of epilepsy may in fact be part of the natural history of any or all of the diseases coming within the province of obstetricians, surgeons or physicians, and to this circumstance is due the many different interpretations placed upon this affection accordingly as it is viewed from one or the other of these standpoints. It is more than probable that undue stress is unconsciously placed upon circumstances which have no other relation to the disease than being familiar facts in the particular province of physicians engaged in special practice. One who has much to do with cases of epilepsy cannot but be forcibly reminded of this by the statements of patients or their friends as to the various opinions received from different physicians as to the cause of the disease in a given case. In no class of cases is this fact more apparent than in those where syphilitic infection either really exists or is supposed to exist. Patients who have ever exposed themselves to venereal disease, as well as those who have contracted gonorrhœa, or ulcers upon the genitals, are quite as apt to have their attention drawn to this mode of developing convulsions, and be classed as cases of syphilitic epilepsy, as those who give a connected and unmistakable history of constitutional infection.

In the causation of epilepsy, as of other forms of nervous disease, we have the important factor of hereditary predisposition. In his remarks on the theory of epilepsy, Dr. Sieveking says:

"If we apply a lighted taper to a muslin curtain, the boarding of a wooden hut, or solid masonry of a church, the effect will vary with the greater or less inflammability of the different structures. The muslin curtain will speedily take fire and flare away; the planks may be scorched, but will probably not inflame; while the stones will show no traces of the influence of the destructive agent which the first shower will not wash away. In the first two instances there is a possibility of ignition; in the third it is not possible. Mankind vary similarly in their tendency to nervous disorders generally and to epilepsy especially; some are utterly insusceptible to influences that may produce them; others, like the wood and the muslin, are more or less impressionable. But wherever the disease occurs it is essentially the same disease; the same symptoms characterize it; it follows the same course, and, unless checked, leads ultimately to the same results."

A more comprehensive state of the case than these few lines convey, could not be obtained. No one becomes epileptic because he or she is a man or a woman, and has arrived at a certain age, however potent age and sex may be in the causation of this complaint. Such circumstances merely concur to aid a predisposition already inherited. In like manner, injuries of the head, nervous exhaustion however induced, diathetic diseases, and reflected irritations, are, in and of themselves, incompetent to produce epilepsy. Nevertheless, they are all-important factors in determining the advent of attacks in persons so predisposed.

I have here grouped together several cases of epilepsy which agree in but one particular: that of acknowledging for their exciting cause the influence of constitutional syphilis. I have rejected many cases in which, while the effects of treatment would incline one to imagine the influence of specific taint, the history was void of those evidences of constitutional infection, such as are related by the patients whose cases I have inserted here. In the same manner, and for a similar reason, no cases have been deemed truly syphilitic from the mere circumstance that the patient had a venereal sore upon his penis at some preceding time. Nothing but a clear and connected account of a primary lesion, and constitutional symptoms, succeeded by the development of epileptiform convulsions, has been deemed sufficient to justify the classification of any case under the head of syphilitic epilepsy.

Case I. In 1868 this patient contracted the initial lesion of syphilis in Philadelphia, and presented the usual symptoms of constitutional infection—cutaneous eruption and falling of the hair

—during the summer of that year. In October, nocturnal pains and nodes on the tibiæ made their appearance, for the relief of which he underwent mercurial treatment. In the spring of 1869, without any premonitory symptoms, he had a number of epileptiform convulsions in rapid succession; since that time he has been subject to fits which make their appearance about once a week. In August, 1869, moved to New York. January 21, 1870, came under my treatment. At that time there were no evidences of syphilis, and the patient complained of no other symptoms indicating disorder of the nervous system than the weekly recurrence of the convulsions. The ophthalmoscope revealed retinal congestion. The treatment adopted was large doses of the bromide of potassium (15 grains three times a-day, dissolved in a wine-glassful of water); the effects of the drug upon the cerebral circulation being carefully watched with the ophthalmoscope. The fits disappeared for an interval of nearly three months. During the latter part of March, nocturnal pains again appeared; in April, the epileptiform convulsions returned, increased in number and altered in character: the loss of consciousness was not so complete as formerly, and occasionally would be but little affected during a paroxysm. The paroxysms were now exclusively nocturnal, and he would at times have as many as a dozen fits in rapid succession. The bromide was suspended for a few days, and the iodide in 20-grain doses, three times a-day, substituted. At the end of a week they were given conjointly, and so continued for a month. Subsequently, the iodide was administered alone, in doses of 10 grains twice a-day. The last fit occurred about April 25, 1870. At the present time (February 20, 1871), this patient reports that he has had no further trouble.

This patient resolutely denied ever having contracted syphilis until many weeks after the treatment directed against the convulsive disorder had been instituted. The account he then gave was so clear and explicit as to leave no doubt of his having had the disease. Prior to the use of the bromide he had been taking the iodide of potassium, for the purpose, as he expressed it, of curing the rheumatic pains from which he suffered. I was unacquainted with these circumstances until he requested permission to resume the iodide, when, upon questioning closely, he told the whole story. During the latter part of March he was absent from the city for a number of days; no anti-syphilitic remedies were employed until after the convulsions returned in April, when the iodide of potassium was used in the manner above described. When last seen, in February, 1871, this patient not only reported that he had been free from the fits, but that his general health was much better than it had been for years.

It is difficult to form a proper estimate of the causes which operated to produce the convulsive seizures of April, 1870. The nature and frequency of the attacks, as well as the time of their occurrence, would seem to indicate that they were at least partially due to the excessive use of the bromide; while the simultaneous occurrence of the peculiar pains of syphilis, after a long interval of freedom, shows that at that particular time the specific poison was again becoming active. This patient was also absent from town for a considerable period prior to their recurrence, and my notes of the ophthalmoscopic appearances are defective at the very time when their evidence would be of service.

Case II. A gentleman, 35 years of age, presented himself in August, 1870, suffering from epileptic paroxysms occurring weekly. The attacks presented no unusual character, and there were no other symptoms referable to the nervous system. His father died of paralysis at the age of 58. During the year 1867 contracted venereal disease in New Orleans, and was treated by a prominent surgeon of that city. Constitutional symptoms came on in two or three months, but were promptly dissipated by treatment. In 1868, nodes came on the shin-bones, and he suffered from nocturnal pains. The iodide of potassium relieved these symptoms, and no further trouble was experienced until July, 1869, when the first epileptic fit came on. His business (that of a commercial traveler) rendering it impossible for him to remain long in one place, he was under no regular treatment for nearly a year. During this time the attacks were exclusively diurnal, and recurred at irregular intervals of from one to two weeks. When I first saw him, in August, 1870, there were a number of swellings on the tibiæ, the remains of former periostitis. A small but exceedingly painful node occupied the left mastoid process, and he complained of nocturnal pains in the region of the left supra-orbital nerve. He was still taking the iodide of potassium in doses of 5 grains twice a-day. He was directed to increase the iodide until 20 grains three times a-day were taken, and to take in addition the bromide in doses of 15 grains before each meal. This latter remedy was only continued a month, but the iodide is still being used. No convulsions since September, 1870.

In this case the syphilitic history was equally clear with the preceding one, and the subsidence of the convulsive phenomena directly due to the specific treatment. The bromide may have relieved any temporary condition of cerebral irritation, but it was doubtless due to the iodide that the patient was freed from his epileptiform symptoms. From the very commencement of the administration of the large doses of the latter remedy (of which smaller quantities had been given for a long time without any beneficial result), the nocturnal pains disappeared, the swelling on the mastoid process subsided, and the fits failed to return. It was the evident connection of the specific symptoms with the convulsions that induced me to stop the bromide at such an early date, and trust to the anti-syphilitic treatment alone. The result has fully justified the action. In January, 1871, this patient exhibited no symptoms of syphilis. He was still taking scruple doses of the iodide.

Case III. A laboring man, residing in Jersey City, presented himself at the Out-Door Department of Bellevue Hospital, suffering from partial paralysis of the right leg. This was in March. 1870. He was 39 years of age, and stated that when 25 years of age he contracted a venereal sore, which, within a few months. was followed by a pustular cutaneous eruption, sore throat, and inflammation of the eyes. For these complaints he was under treatment fully a year, but was finally discharged, cured, as he imagined. The next difficulty he experienced was a very unsightly swelling of the nose, with a profuse discharge of offensive pus, severe nocturnal pains, and almost complete deafness. This was three years after the primary sore. The physician who treated him for these last symptoms prescribed large doses of the iodide of potassium, with the effect of greatly relieving him. In the year 1860 he came to the United States, and was free from all evidence of the disease, except slight deafness, for four years. In the latter part of 1862, while serving as an army teamster, he observed that the ear most affected was the seat of a peculiar and unusual sensation. Without any premonitions, he would suddenly hear a buzzing noise in that ear (the right one), with a sensation of burning, affecting the whole organ, and a tickling, tingling feeling deep in the meatus. In the beginning, these attacks were so slight as to scarcely attract his attention. As time elapsed, however, they became much more severe. Instead of the subjective noises, with itching and tingling, these attacks manifested themselves by a sudden, loud report in that ear, with momentary vertigo, followed by a great desire to rub that side of his head. The number of these attacks increased, and some were much more severe than others. In some he became dizzy and blind, but still retained consciousness sufficiently to be acutely sensible of the disagreeable feelings in his ear; while in others he could remember nothing beyond the sudden loud report which always preceded an attack—for the ensuing moment he would be unconscious. He still retained his team and attended to his ordinary business until July, 1864, when, after one of these ear-attacks, he had a fully-developed epileptic fit. During this month nocturnal pains also made their appearance, and caused him so much trouble that he resigned his employment, came to New York, and underwent specific treatment. He had about fifteen fits in all, extending over a period of two months. They were always preceded by the peculiar ear-symptoms above described. Treatment promptly dissipated the convulsions, nocturnal pains, and ear-troubles.

The partial paralysis of the right leg was relieved by large doses of the iodide of potassium (20 grains, three times a-day) conjoined with hypodermic injections of strychnia.

Although the epileptiform symptoms in this case were developed six years ago, and the whole number of attacks was very small, yet I have related the history at some length, owing to the exceptionally clear account given of the manner in which the aura was developed, and the evident relationship between it, the milder forms of the disease which occurred early, the fully developed paroxysm which occurred latter, and those evidences of constitutional infection which were present at all times.

CASE IV. A railroad conductor, aged 43, contracted constitutional syphilis in 1864. During the winter of 1866–7 was troubled with painful swellings of the clavicle and bones of the head, with diffused pains in his limbs, worse at night, for the relief of which he took the iodide of potassium. In December, 1869, had an attack of acute mania, succeeded by external strabismus and slight facial paralysis. His wife remembers that for several months previous to this he had been troubled with fits of dizziness and momentary absence of mind, recurring several times a-day. In March, 1870, epileptic attacks, nocturnal in character, made their appearance; returning at intervals of from four to six weeks, accompanied by daily attacks of the *petit mal.* During the summer he gradually lost power in the lower extremities, and suffered from boring pains in the spine. In November, 1870, was exposed for a length of time to severe cold, and from that time his symptoms increased so much in severity that he was compelled to take to his bed early in December. The epileptic attacks now became diurnal in character, and recurred several times a-day. He emaciated rapidly, and suffered much from insomnia and depression of spirits. Acute atrophy of the pronator and flexor muscles of both forearms also made its appearance, and progressed with great rapidity. On the occasion when I first saw this patient, January 1, 1871, he was almost imbecile; had paralysis of the left side of the face, and of the muscles supplied by the left third cranial nerve; anæsthesia of the forearms and legs, and atrophy of the thenar eminences of both hands and the pronators and flexors of both forearms. The fits were recurring twice a day. The ophthalmoscope revealed intense congestion of the retinal vessels, but no organic changes in the papillæ. Bed-sores had formed on both hips, and the left ankle was the seat of a syphilitic ulcer of long duration. Three large and very painful nodes were discovered on the occipital bone, while clavicles and tibiæ exhibited evidences of old periostitis. This patient was placed upon 10-grain doses of the iodide of potassium, three times a-day. These were persisted in for about a week, and then it was so arranged that the dose could be increased one grain daily. The bromide, in doses of thirty grains, was also administered each evening. These, in connection with sustaining diet and a proper bed, were all the remedial measures adopted. By the end of January, the dose of the iodide having increased to 30 grains, it was kept at that quantity, and at the present time (February 23) is still being administered.

This patient is still under observation. Since the commencement of treatment, the epileptiform symptoms have disappeared, the mental powers have improved, and the patient so far relieved as to be able to leave his bed. The case is noteworthy as regards the prompt relief of the former. But little efficacy is, I think, to be ascribed to the bromide, for equally good results would doubtless have been obtained from the employment of sufficiently large doses of the iodide alone. It is also to be remembered that, owing to the recent date at which the epileptiform symptoms were developed, a sufficient length of time had not elapsed to allow the system to acquire the epileptic habit.

Case V. In the case of J. C., aged 37, syphilis was contracted in 1853, and proved more or less troublesome for two years. During this time all the symptoms were characteristic of constitutional infection, and he was relieved by specific treatment. In June, 1855, had his first epileptic fit. The paroxysms returned again in 1856, and gradually became more frequent. He was a soldier during the Rebellion, and although his fits came on about once a month, he found no difficulty in performing all his duties. In December, 1869, when I first saw him, the fits were manifesting themselves two or three times a-week, and recurred indifferently by day or night. At this time he was also suffering from necrosis of the right tibia. The ophthalmoscopic appearances indicated hyperæmia of the disk and retina. There were traces of old iritis in the left eye.

I have already called attention to this case in an article which appeared in the *New York Medical Journal* for February, 1871, as an illustration of the value of the ophthalmoscope in the treatment of cases of epilepsy. This was clearly shown in the treatment of this patient. As is above stated, the ophthalmoscopic appearances indicated cerebral hyperæmia, and in pursuance of the plan I commonly adopt in cases exhibiting these appearances, I prescribed the bromide of potassium in doses of 15 grains, three times a-day, and directed the patient to report twice a-week for examination. In neither of these respects were my directions complied with. He passed from under my observation for nearly three weeks, and when he came back, it was to report a return of the convulsions. Instead of taking a teaspoonful of the solution ordered (equivalent to 15 grains of the drug), he took twice and thrice that quantity. As a consequence, the paroxysms at once ceased with the relief of the cerebral hyperæmia, but when the physiological effect of the remedy was carried too far, an anæmic condition was induced, and from this widely opposite anatomical condition the paroxysms returned with greater violence than ever. This case also illustrates the fact that success in the treatment of epilepsy depends much less upon the particular drug prescribed than upon the care and skill employed in regulating the administration of the remedy selected. In the present case, the presence of this anæmic condition was plainly evident from an examination with the ophthalmoscope. To remedy it, a drug was employed which experience has demonstrated to possess the power of increasing the amount of blood circulating through the cerebral blood-vessels—the sulphate of strychnia. This was administered in the form of a solution (2 grains to an ounce of water), in doses of ten drops, three times a-day; and very soon after its administration was commenced, the convulsions again disappeared. The patient was carefully watched from that time forward, and the bromide of potassium or sulphate of strychnia were used just as they were indicated by the varying condition of the blood-supply of the brain. His last fit occured in January, 1870.

In the above case we have an illustration of the fact that epilepsy, primarly developed by syphilis, may continue and grow worse from the influence of habit alone. It is true that this patient was suffering from necrosis at the time he came under my observation, and it is probable that the specific taint may have been instrumental in its development; yet the fact that the epilepsy was so affected by remedies, none of which were anti-syphilitic, as to produce a supension of the paroxysms for fifteen months, is strong evidence that no active influence was exerted by the syphilitic poison. Had the latter been present in an active form, no such results could have been obtained by the treatment adopted.

CASE VI. R. O'G., a laborer, native of Ireland, aged 43 years, contracted syphilis in August, 1870, and had a copper-colored eruption, alopecia, sore throat, and double vision within ten weeks. Subsequently he suffered from nocturnal pains in his limbs and nodes on the tibiæ. In December, 1870, after a brief attack of localized pain in the head, had a convulsive seizure. These fits recurred at the rate of eight or ten a-week. In the intervals between paroxysms complained of double vision and vertigo. Came under treatment January 9, 1871. Was ordered—

℞.—Hydrargyri Bichloridi, - - - - grs. j.

Potassii Iodidi, - - - - - - ℥ ss.

Aquæ ad., - - - - - - - ℥ iv. M.

Dose, a teaspoonful in a wine-glass of water after each meal. At the same time he was to live generously, and take plenty of out-door exercise. When this patient was last seen he was still taking the above prescription. He reported that no fit had occurred since treatment was commenced, and that the vertigo and diplopia had completely disappeared. Still under observation.

In the preceding cases the interval between the contraction of the primary sore and the supervention of the convulsions varied from five months to eight years. In other examples of syphilitic epilepsy which have come under my observation the discrepancy is equally great. It is probable that the particular time at which, in given cases, epileptiform symptoms are developed is explicable only upon the same hypothesis which it is necessary to resort to in order to account for the development of epilepsy in some, and not in all, cases of syphilis. The "unknown element" which we have to postulate is the variable factor of hereditary predisposition; and the only explanation we can obtain is that in proportion to the strength of the predisposition the patient will have the convulsions occurring early in the course of the syphilitic disease. In other words, the stronger and more efficient the predisposition, the slighter will be the exciting cause necessary to develop the complaint.

The diagnosis of syphilitic epilepsy is as exact and satisfactory in certain cases as it is uncertain and tentative in others. In all cases it rests upon our ability to attain a complete syphilitic history, to discern evidences of the persistence of the disease in the patient under examination, and upon the results of specific treatment. No phenomenon of epilepsy, not connected with the above circumstances, can be considered pathognomonic. Cases in which a syphilitic taint can be excluded, will be found to present all the appearances relied upon by some to show a specific causation. Local paralysis, the rapid succession of attacks with long intervals of freedom, nocturnal paroxysms—in fact, any or all the symptoms occasionally or generally present in syphilitic epilepsy, will be found equally common, not only in cases which present no evidences of specific disease either by history or personal examination, but in individuals who have been subjected to prolonged anti-syphilitic treatment. And further, these same cases will have their unfortunate condition ameliorated, and in some instances be completely cured, by methods of treatment purely anti-epileptic.

The treatment of cases of epilepsy due to syphilitic poisoning depends mainly upon the length of time the patient has been subject to convulsions and the presence of evidence of syphilitic disease. The effects of specific remedies will vary greatly, as regards their power to control the convulsions, with the former circumstance. When the convulsive seizures are of recent date, and but few attacks have occurred, the iodide of potassium, either singly or combined with some form of mercury, will prove sufficient not only to dissipate the syphilitic symptoms, but to cure the epilepsy. If the convulsions have recurred frequently, and been present for a length of time, anti-syphilitic remedies alone will, in the vast majority of cases, prove ineffectual. With such, it is necessary to resort to measures especially adapted to prevent the recurrence of the paroxysms. The epileptic habit then becomes an important element, and attention must be directed to breaking it up. That provision of the nervous system by which voluntary acts become more and more easy with each repetition, and finally reach that stage in which they can be performed unconsciously, appears also to be in active operation in the same manner, to accustom the system to involuntary and unconscious acts of pathological origin. It is a matter of observation that convulsive seizures, excited by evanescent causes, after a certain amount of repetition, persist apparently from the force of habit long after their original cause has disappeared, and with each recurrence not only become more easily excited, but grow more inveterate. In such cases curative measures must be directed to the eradication of this convulsive tendency—this epileptic habit.

For this reason, the treatment of each individual case of epilepsy becomes a matter for special study. The general health of the patient must be carefully looked after, and if any error of nutrition is present, appropriate remedies must be prescribed. In any case where there is even the faintest reason to suspect the presence of syphilitic taint, it is justifiable to resort to anti-syphilitic remedies, and give the patient the benefit of the doubt. No possible harm can result from a trial of the iodide of potassium, and where there is any difficulty of this nature, the benefit will be at once apparent.

In an article published in the February number of the *New York Medical Journal*, I briefly referred to the value of the ophthalmoscope as a means of determining the immediate cause of the paroxysms in cases of epilepsy accompanied by derangements of the cerebral circulation. I am fully convinced that the persistent and careful observation of the brain-circulation, as revealed by changes in the blood-supply of the retina, not only affords an explanation of many otherwise obscure phenomena of the disease, but affords a ready means of determining the effects of remedies.

In those cases where the evidences of specific disease are doubtful, and no favorable result has been obtained from anti-syphilitic treatment, as also in any case of epilepsy in which we are compelled to resort to measures intended to control the paroxysms simply, the ophthalmoscopic appearances afford rational indications for therapeutical procedure. It is unnecessary to dwell upon the well-known fact that those widely opposite conditions—congestion and anæmia of the brain—are equally competent to produce convulsive phenomena. Physiological experiments have not only demonstrated that fact, but they lead us to infer that those many and widely diverse bodily conditions which induce this disease, do so by altering the quantity and quality of the blood circulating through the brain. There is but one method by which we can determine the condition of the cerebral circulation with any approach to accuracy, and that is by the employment of the ophthalmoscope. Congestion of the cerebral structures is then known by the presence of evidences of overfulness of the retinal vessels, while the opposite condition of the intra-ocular structures indicates intra-cranial anæmia. These two opposite conditions are distinctive of two classes of epileptics, and afford rational indications for treatment. The one class will be benefited and the other injured by the ordinary plan of administering large doses of bromide of potassium without regard to other circumstances than the fact that the individual has epilepsy.

In regulating measures of treatment calculated to break up the epileptic habit, this plan will prove of great service. While with appropriate constitutional treatment the tissue-changes which primarily induced the disease can be removed, these examinations, by giving information of the state of the cerebral circulation, will enable us to avoid producing any undue effect with our remedies, and so keep the paroxysms in abeyance until a sufficient length of time has elapsed to allow the nervous system to recover its healthy tone. This will of course vary with different cases; but with those in which a specific taint is the exciting cause, I think it can be confidently asserted that, along with constitutional measures, any course of procedure which will keep the paroxysms in abeyance for a year, will eradicate the epileptic tendency. I have yet to meet with a case of epilepsy, of any description, in which the paroxysms have returned after such a course had been adopted for that length of time.

